# KA1-targeted regulatory domain mutations activate Chk1 in the absence of DNA damage

**DOI:** 10.1038/srep10856

**Published:** 2015-06-03

**Authors:** Eun-Yeung Gong, Veronique A. J. Smits, Felipe Fumagallo, Desiree Piscitello, Nick Morrice, Raimundo Freire, David A. Gillespie

**Affiliations:** 1 Instituto de Tecnologías Biomédicas, Centro de Investigaciones Biomédicas de Canarias, Facultad de Medicina, Campus Ciencias de la Salud, Universidad de La Laguna, La Laguna 38071, Tenerife, Spain; 2 Unidad de Investigación, Hospital Universitario de Canarias, Instituto de Tecnologías Biomédicas, Ofra s/n, La Cuesta, La Laguna 38320, Tenerife, Spain; 3 Beatson Institute for Cancer Research, Garscube Estate, Switchback Road, Glasgow G61 1BD, U.K

## Abstract

The Chk1 protein kinase is activated in response to DNA damage through ATR-mediated phosphorylation at multiple serine-glutamine (SQ) residues within the C-terminal regulatory domain, however the molecular mechanism is not understood. Modelling indicates a high probability that this region of Chk1 contains a kinase-associated 1 (KA1) domain, a small, compact protein fold found in multiple protein kinases including SOS2, AMPK and MARK3. We introduced mutations into Chk1 designed to disrupt specific structural elements of the predicted KA1 domain. Remarkably, six of seven Chk1 KA1 mutants exhibit constitutive biological activity (Chk1-CA) in the absence of DNA damage, profoundly arresting cells in G2 phase of the cell cycle. Cell cycle arrest induced by selected Chk1-CA mutants depends on kinase catalytic activity, which is increased several-fold compared to wild-type, however phosphorylation of the key ATR regulatory site serine 345 (S345) is not required. Thus, mutations targeting the putative Chk1 KA1 domain confer constitutive biological activity by circumventing the need for ATR-mediated positive regulatory phosphorylation.

The Chk1 protein kinase is activated in response to damaged DNA and stalled replication forks and acts as a central effector of the DNA damage and replication checkpoint responses in vertebrate cells^1^. Activation of Chk1 depends on phosphorylation of multiple SQ residues within the C-terminal regulatory domain. Serine 345 (S345) in particular is crucial, as several studies have shown that Chk1 mutants bearing non-phosphorylatable alanine residues at this position are biologically non-functional^2^,^3^,^4^. Despite its importance the functional consequences of S345 phosphorylation that lead to Chk1 activation are unknown. Previous studies have associated this modification with release from chromatin^5^, increased ubiquitylation^6^, and binding of 14-3-3 proteins^7^, however exactly how these processes relate to catalytic and biological activity remains unclear.

Structural characterisation has shown that a recombinant Chk1 kinase domain adopts an active configuration when expressed in isolation^8^, indicating that activation loop modification is unlikely to play a role in Chk1 regulation. Furthermore, it has been shown that the C-terminal regulatory domain can bind to the kinase domain^9^,^10^, presumably normally via an intramolecular interaction, and that this interaction can inhibit kinase catalytic activity *in vitro*^8^. One possibility therefore is that ATR-mediated phosphorylation of S345 acts to relieve this inhibitory intramolecular interaction, however exactly how this might be achieved is unclear. A further perplexing observation is that truncation mutants of Chk1 lacking the C-terminal regulatory domain are biologically non-functional^11^, even though they may acquire enhanced catalytic activity, indicating that this region embodies positive function(s) in addition to its potential auto-inhibitory activity^12^.

A major obstacle to understanding the molecular mechanism of Chk1 regulation has been the near-complete absence of information regarding the structure of the C-terminal regulatory domain. Recently however it has been proposed that the Chk1 C-terminal regulatory domain may contain a KA1 domain^12^, a small compact protein fold identified in multiple protein kinases including SOS2 in plants^13^, AMPK^14^ and MARK3^15^ in humans, and Kcc4p, Gin4p, and Hsl1p in budding yeast^16^. The function(s) of KA1 domains are not well understood at present, however they appear to be widely distributed in multiple protein kinases with diverse biological roles and mechanisms of regulation^17^.

Here, we find that the putative Chk1 KA1 domain is predicted to be most similar to the KA1 domain of the plant stress kinase SOS2^13^. We then use this information to assess the functional significance of the predicted Chk1 KA1 structure by introducing tailored point mutations designed to disrupt specific structural elements within the core domain. Remarkably, six out of seven of such Chk1 mutants exhibit constitutive biological activity which depends on kinase catalytic activity but, in marked contrast to wild-type Chk1, no longer depends on ATR-mediated phosphorylation of the key regulatory site, S345.

## Results

### Mutational targeting of the putative Chk1 KA1 domain

**Figure 1:**
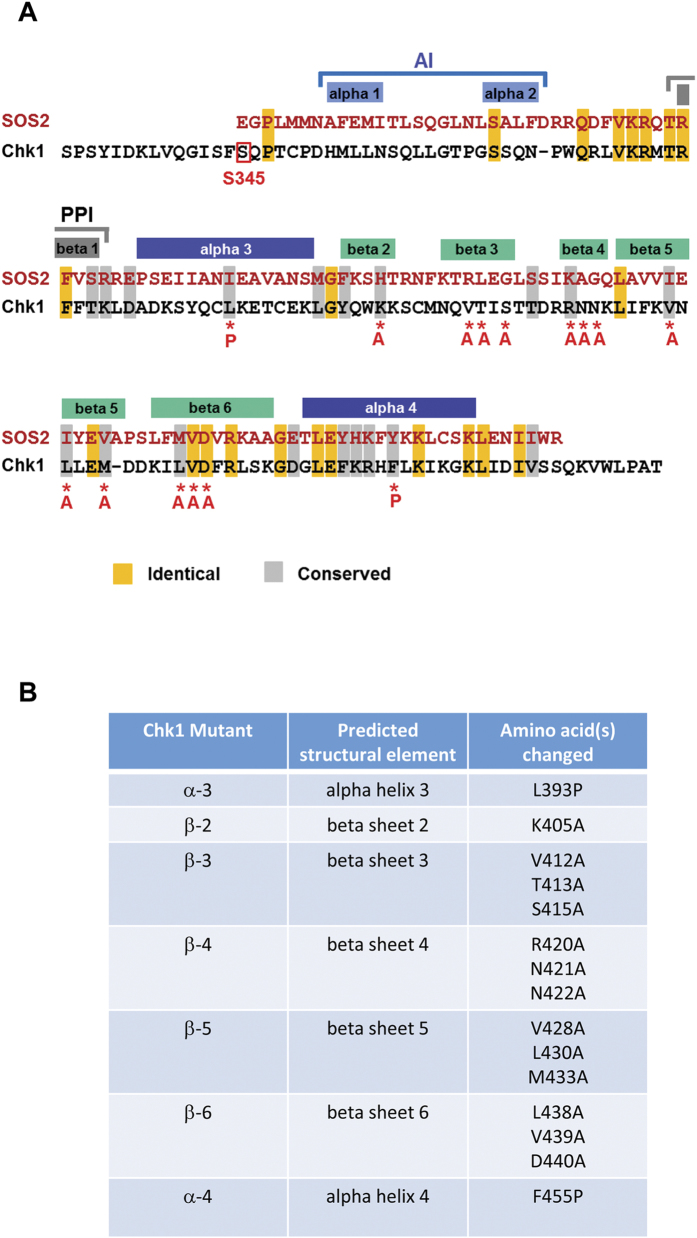
Comparison of the C-terminal regulatory domains of Chk1 and SOS2. **A**) The computer algorithm FUGUE^18^ was used to query the Protein Database (PDB) with the amino acid sequence of the C-terminal regulatory domain of human Chk1 (amino acids 281– 476) to search for potential structural features. The C-terminal KA1 domain of the plant stress kinase SOS2 returned the top Z-score (Z = 19, where >6 is considered to be “certain”). In SOS2 the KA1 domain is preceded by an auto-inhibitory (AI) region and a PP2C-family phosphatase-interacting motif (PPI). These elements were included in the FUGUE alignment with Chk1 and contributed to the overall Z-score. The structurally determined location of the AI, PPI, and the two α-helices and five β-sheets that form the core KA1 fold of SOS2 is indicated above the aligned sequences, whilst the locations of the mutations created in Chk1 is shown below. Amino acid identities and similarities between the C-terminal regulatory domains of Chk1 and SOS2 as originally described^19^ are shown by shading. The location of the essential serine 345 (S345) ATR phosphorylation site in Chk1 is boxed in red. **B**) Table of Chk1 KA1-targeted mutations.

To gain insight into potential structural features in the Chk1 C-terminal regulatory domain, we used the algorithm FUGUE to analyse amino acids 281– 476 of human Chk1^18^. FUGUE compares a query sequence with known protein structures in the Protein Database (PDB) to identify conserved sequences of amino acids with similar chemical characteristics (rather than amino acid identity). This analysis retrieved 3 highly significant hits corresponding to the KA1 domains of the protein kinases SOS2 (Z = 19;^13^), AMPK (Z = 11;^14^), and MARK3 (Z = 9.5;^15^). Of these, the putative KA1 domain of Chk1 was predicted to be most similar to that of the plant stress kinase SOS2^13^. Interestingly, significant amino acid identity and similarity between the regulatory domains of SOS2 and Chk1 was reported previously (Fig. 1A;^19^).

KA1 domains typically consist of five β-sheets and two short α-helices^16^. In the case of SOS2^13^ the core KA1 domain is preceded by an auto-inhibitory region (AI) and a short β-sheet containing a PP2C-family phosphatase interacting motif (PPI). As shown in Fig. 1A, alignment with the determined structure of the SOS2 KA1 domain allowed us to predict the location of the core KA1 structural elements in Chk1 and to target them using site-directed mutagenesis. In order to disrupt the predicted α-helices, and thus potentially destabilise the overall KA1 fold, we introduced helix-breaking proline (P) residues into the middle of each helix to generate mutants Chk1 α-3 and Chk1 α-4 (Fig. 1A, B). Reasoning that the KA1 domain β-sheets might participate in protein-protein interactions, we used multiple species comparison of Chk1 homologues to identify the most highly conserved hydrophobic and charged residues within each predicted β-sheet and then replaced these with alanine (A) to generate mutants Chk1 β-2, Chk1 β-3, Chk1 β-4, Chk1 β-5, and Chk1 β-6 (Fig. 1A, B).

**Figure 2:**
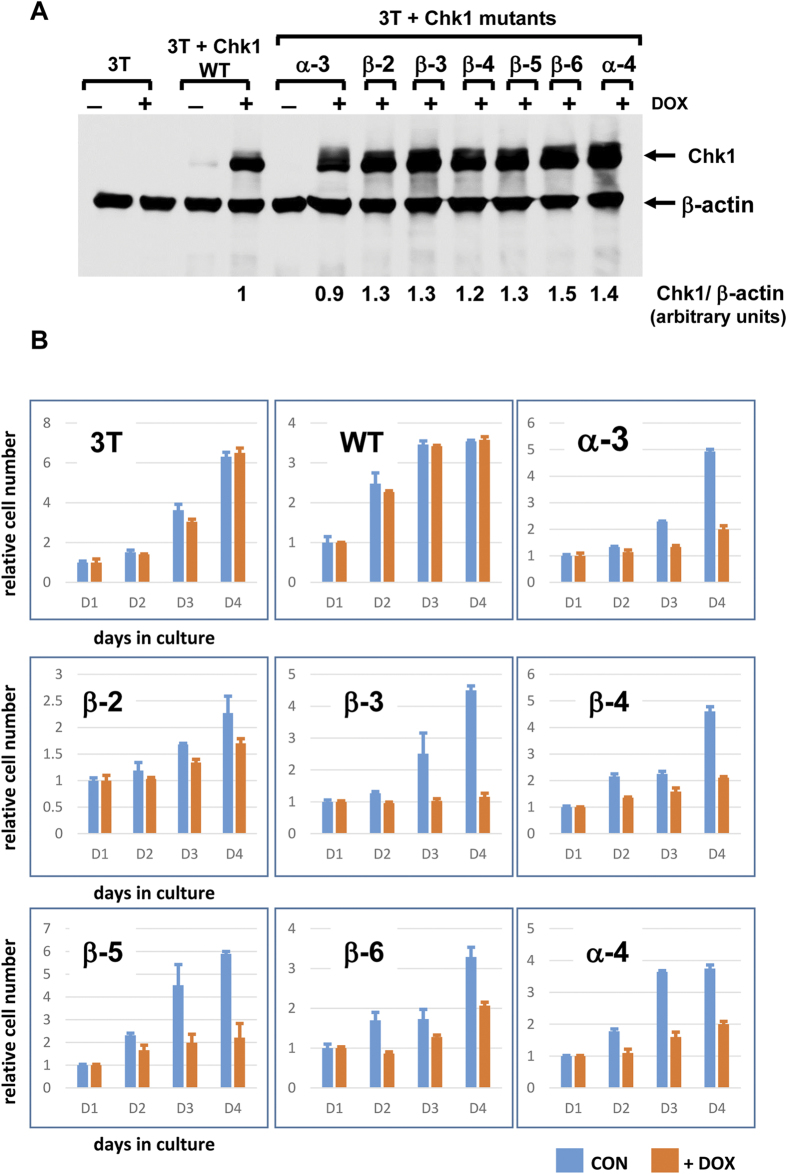
Chk1 KA1 mutants inhibit cell proliferation. **A**) Doxycycline (DOX)-regulated expression of Chk1 WT and the KA1 domain-targeted mutants in Chk1-deficient 3T cells. Cell cultures were treated with DOX (50 ng/ml) or solvent control for 16 hours prior to harvest and analysis of Chk1 protein expression. Expression in the presence and absence of DOX is shown for the negative control (3T), Chk1 WT, and Chk1 α-3, however DOX-dependent expression of all other mutants was confirmed in additional experiments (Supplementary Fig. 1A). β-actin serves as loading control. The levels of exogenous WT and mutant Chk1 protein expression were approximately 3-5 times greater than the level of endogenous Chk1 in wild-type DT40 cells (Supplementary Fig. 1A). **B**) 3T cell cultures expressing Chk1 WT or the indicated mutants were cultured in the presence or absence of DOX (50 ng/ml) for 4 days and cell number quantified each day. The experiment was performed in triplicate, shown is the mean and standard deviation.

The resulting Chk1 mutants were then introduced into a Chk1-knockout DT40 cell line for functional analysis as described previously^3^. Because it seemed possible that at least some of these mutants might acquire constitutive biological activity, and thus potentially be incompatible with cell proliferation, we took the precaution of placing the mutant proteins under the control of a TET-regulated gene promoter so that they would be expressed only when cells were treated with doxycycline (see methods for details). As controls we used the parent Chk1-knockout cell line (3T) and cells expressing inducible wild-type Chk1 (WT). As shown in Fig. 2A, doxycycline (DOX) treatment induced the expression of comparable levels of Chk1 WT and each of the KA1 mutant proteins.

### KA1-targeted mutations activate Chk1 biological function

**Figure 3:**
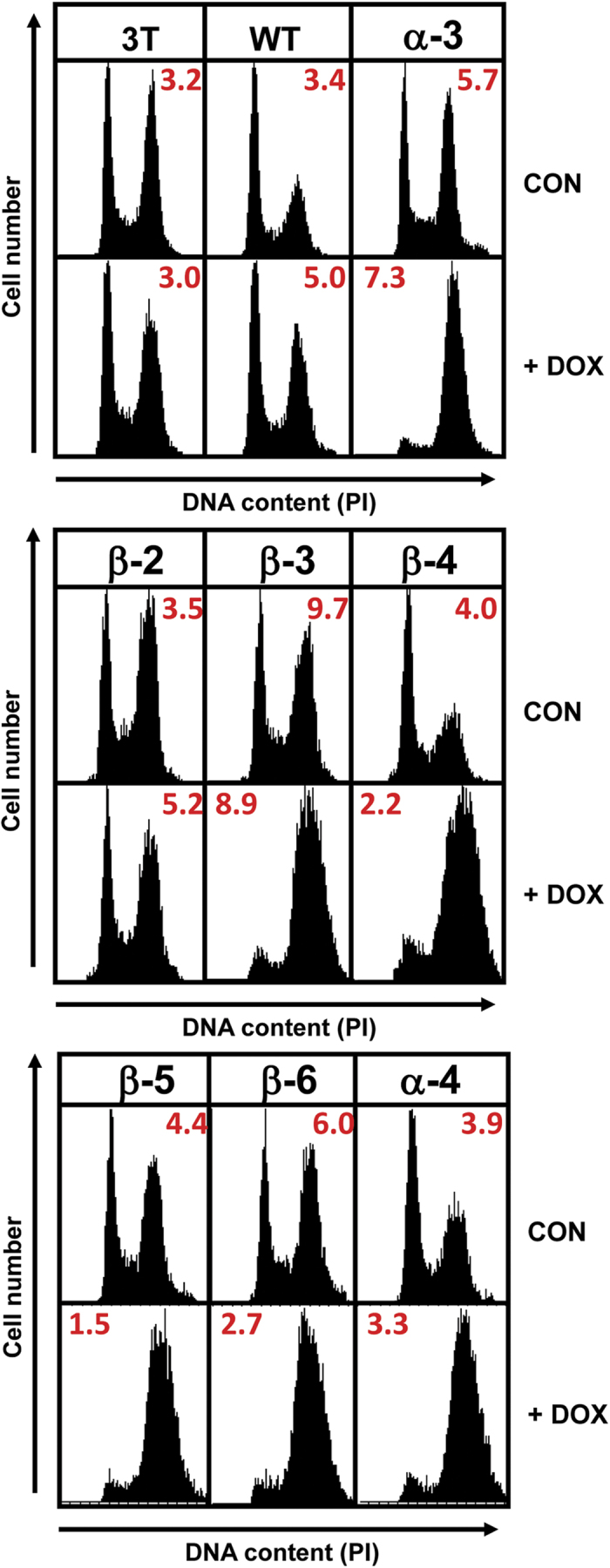
Chk1 KA1 mutants induce cell cycle arrest in G2. 3T cell cultures expressing Chk1 WT or the indicated mutants were treated for 16 hours with DOX or vehicle control, harvested, and analysed by flow cytometry. DNA content histograms are shown together with the mitotic index values as determined by staining for phospho-serine 10 histone H3 shown inset in red. A complete analysis of individual cell cycle phases is presented in Supplementary Fig. 2. The results shown are representative of at least 3 independent experiments.

To investigate the effects of KA1 mutations on Chk1 function, we first determined if their expression affected cell proliferation by culturing cells for 4 days in the presence or absence of DOX. Whereas DOX had no effect on the growth of 3T control cells or cells expressing inducible WT Chk1, the proliferation of cells expressing all of the KA1 mutants was diminished in the presence of DOX (Fig. 2B). The scale of this effect varied from almost complete growth inhibition (eg. Chk1 α-3, β-3) to a more modest slowing of cell proliferation (eg. Chk1 β-2). To understand the basis for this growth inhibition, we induced the expression of WT and mutant forms of Chk1 for 16 hours and examined the cell cycle distribution of the DOX-treated and control cultures by flow cytometry. As shown in Fig. 3, DOX treatment had little or no effect on the cell cycle distribution of the 3T cell line or cells expressing WT Chk1 (also see Supplementary Fig. 2). Remarkably however, expression of all of the Chk1 KA1 mutants with the exception of Chk1 β-2 resulted in a dramatic change in the cell cycle distribution of the DOX-treated cultures, with a majority of cells accumulated in G2/M phase of the cell cycle (Fig. 3 and Supplementary Fig. 2). Because the great majority (>90%) of these cells were negative for the mitotic marker phospho-serine 10 histone H3 (pH3; Fig. 3), it was evident that they were accumulated in G2 rather than mitosis. As expected from previous work^20^, we also observed an increase in inhibitory Cdk1 tyrosine 15 (Y15) phosphorylation in the arrested cells, an established target of the G2 checkpoint (Supplementary Fig. 1B). We saw no evidence for any increase in cells with sub-G1 DNA content, arguing against cell death as a significant cause of growth inhibition.

Taken together, these data demonstrate that six of the seven Chk1 KA1 mutants we generated potently inhibit cell proliferation by arresting otherwise unperturbed cells in the G2 phase of the cell cycle. Since Chk1 normally mediates G2 arrest only under conditions of DNA damage when it is activated by ATR, we conclude that these mutants have acquired constitutive biological activity (Chk1-CA mutants).

### Chk1-CA mutants exhibit enhanced catalytic activity

We next asked if active Chk1-CA mutants exhibited increased kinase activity. WT and mutant Chk1 protein expression was induced with DOX then the proteins were immunoprecipitated and tested for kinase activity using a non-radioactive assay employing recombinant GST-CDC25C as a substrate. In this assay Chk1-mediated GST-CDC25C serine 216 (p216) phosphorylation, a validated Chk1 target site^21^,^22^, is monitored by western blotting using a phospho-specific antibody recognising pS216. Kinase reactions prepared with the immunoprecipitated Chk1 WT or mutant proteins were sampled after 10 and 30 minutes to ensure that substrate saturation was not reached (see methods for details).

**Figure 4:**
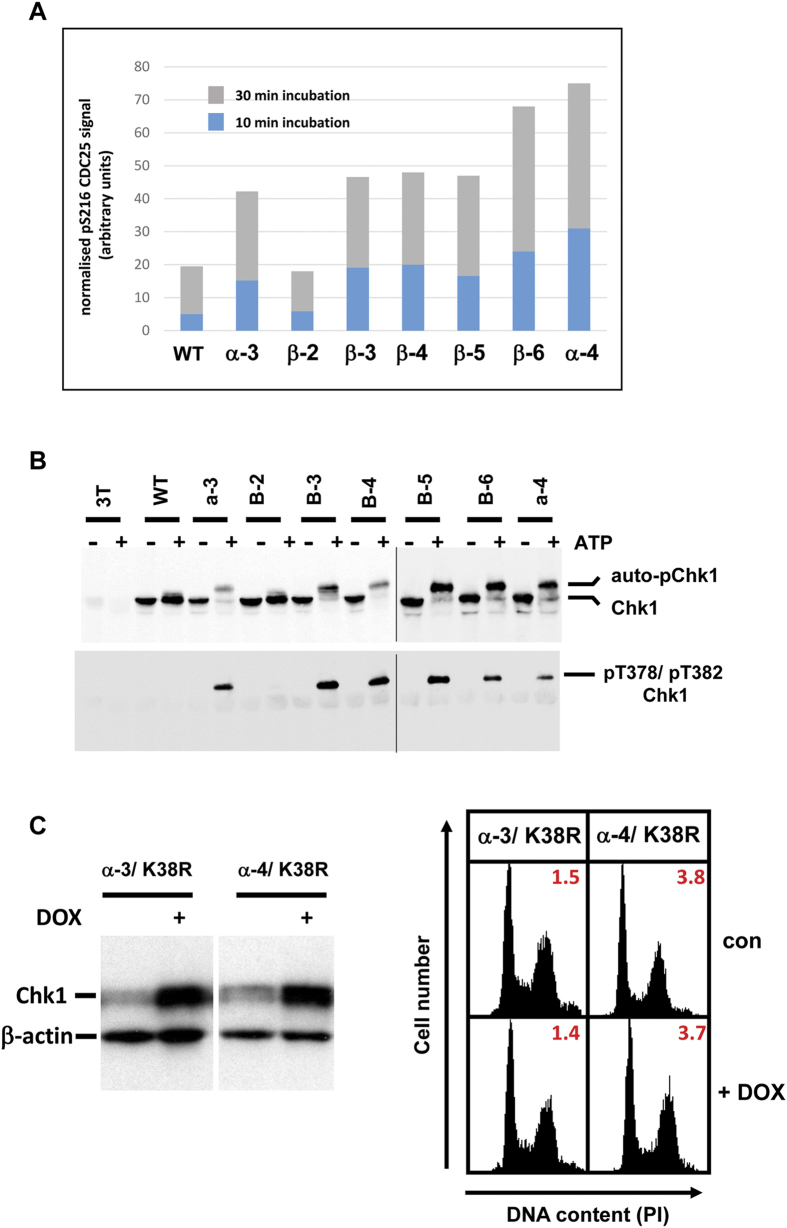
Biologically active Chk1 KA1 mutants exhibit enhanced catalytic activity. **A**) 3T cell cultures expressing Chk1 WT or the indicated mutants were treated for 16 hours with DOX to induce Chk1 expression, lysed, and immunoprecipitated using anti-Chk1 antibody. A kinase assay was performed with the washed immunoprecipitates using GST-CDC25 as a substrate. Substrate phosphorylation was measured using a phospho-specific antibody against pS216 in CDC25, a validated Chk1 phosphorylation site, by western blotting of the kinase reactions. Kinase reactions were sampled at 10 and 30 minutes to ensure that substrate saturation was not reached during the assay. The results shown are representative of 3 independent experiments. See methods for further details. **B**) 3T cell cultures expressing Chk1 WT or the indicated mutants were treated for 16 hours with DOX to induce Chk1 expression, lysed, and immunoprecipitated using anti-Chk1 antibody. The resulting immunoprecipitates were resuspended in kinase buffer, divided in two, and incubated in the presence or absence of ATP for 30 minutes at 30 °C. Samples were then analysed by western blotting using anti-total Chk1 or a phospho-specific antiserum specific for Chk1 phosphorylated on threonine 378 and threonine 382 (T378/T382 – see methods for details). **C**) Secondary mutations of lysine 38, which is essential for Chk1 catalytic activity, to arginine (K38R) were introduced into Chk1 α-3 and α-4 to generate the double mutants Chk1 α-3/K38R and Chk1 α-4/K38R. 3T cell lines expressing each mutant were treated with DOX (50 ng/ml) or vehicle control for 16 hours, harvested, and analysed by western blotting (left panel) or flow cytometry for DNA content and mitotic index (right panel). A complete analysis of individual cell cycle phases is presented in Supplementary Fig. 3A.

As shown in Fig. 4A, this analysis revealed that, with the exception of Chk1 β-2, all of the other Chk1-CA mutants exhibited basal levels of kinase activity that were between 2-6 fold greater than WT. In comparison the basal kinase activity of Chk1 β-2, which had little or no effect on cell cycle distribution in the short term (Fig. 2C), was indistinguishable from Chk1 WT. These data demonstrate that there is a close correlation between increased basal kinase activity of Chk1-CA mutants and cell cycle arrest.

Chk1 is also known to undergo auto-phosphorylation^23^,^24^ and we investigated whether this property was altered by the KA1 mutations. WT and mutant Chk1 proteins were induced with DOX, immunoprecipitated, and then incubated in a kinase reaction with or without ATP. Samples were then analysed by western blotting for the modification state of the immunoprecipitated Chk1 proteins. As shown in Fig. 4B, this analysis revealed that all of the Chk1-CA mutants rapidly underwent auto-phosphorylation in the presence of ATP that resulted in a pronounced gel-shift whereas Chk1 WT and Chk1 β-2 did not. We also examined the modification of two specific sites of auto-phosphorylation that we have recently identified, threonine 378 and threonine 382 (T378/T382), using a phospho-specific antibody. The mapping of T378/T382 as sites of auto-phosphorylation and the characterisation of this antibody will be described in detail elsewhere, however analysis of the kinase reactions indicated strong modification of these sites in Chk1-CA mutants but not in Chk1 WT and Chk1 β-2 (Fig. 4B). We conclude that KA1 mutations that confer constitutive biological activity on Chk1 enhance basal catalytic activity against exogenous substrates and also markedly stimulate the rate of spontaneous auto-phosphorylation observed *in vitro*.

To determine if constitutive biological activity was dependent on kinase activity, we introduced a secondary mutation of lysine 38 to arginine (K38R), which has previously been shown to inactivate Chk1 kinase catalytic activity^3^, into the Chk1 α-3 and α-4 mutants. The resulting double mutants were introduced into 3T cells and we investigated their effect on cell cycle distribution after induction with DOX (Fig. 4C). In marked contrast to their single mutant parents (Fig. 3), expression of the double mutants α-3/K38R and α-4/K38R had no measureable effect on cell cycle distribution (Fig. 4C, Supplementary Fig. 3A), indicating that cell cycle arrest induced by the Chk1 α-3 and α-4 mutants depends on kinase catalytic activity.

### Chk1-CA mutant activity is independent of ATR-mediated positive regulatory S345 phosphorylation

ATR-mediated phosphorylation of S345 is normally essential for Chk1 activation in response to DNA damage and replication stress^2^,^3^,^4^. We therefore considered that the Chk1-CA mutants might either exhibit increased or constitutive levels of this modification in the absence of damage, or alternatively, no longer require this modification for their biological activity. To distinguish between these possibilities we first compared the basal and DNA damage-induced levels of S345 phosphorylation of WT Chk1 with the Chk1-CA mutant proteins. 3T cell lines expressing WT Chk1 and the various KA1 mutants were induced with DOX for 16 hours and then treated with etoposide for a further 6 hours to induce DNA damage. Samples were then analysed by western blotting using an antibody specific for Chk1 phosphorylated on S345.

**Figure 5:**
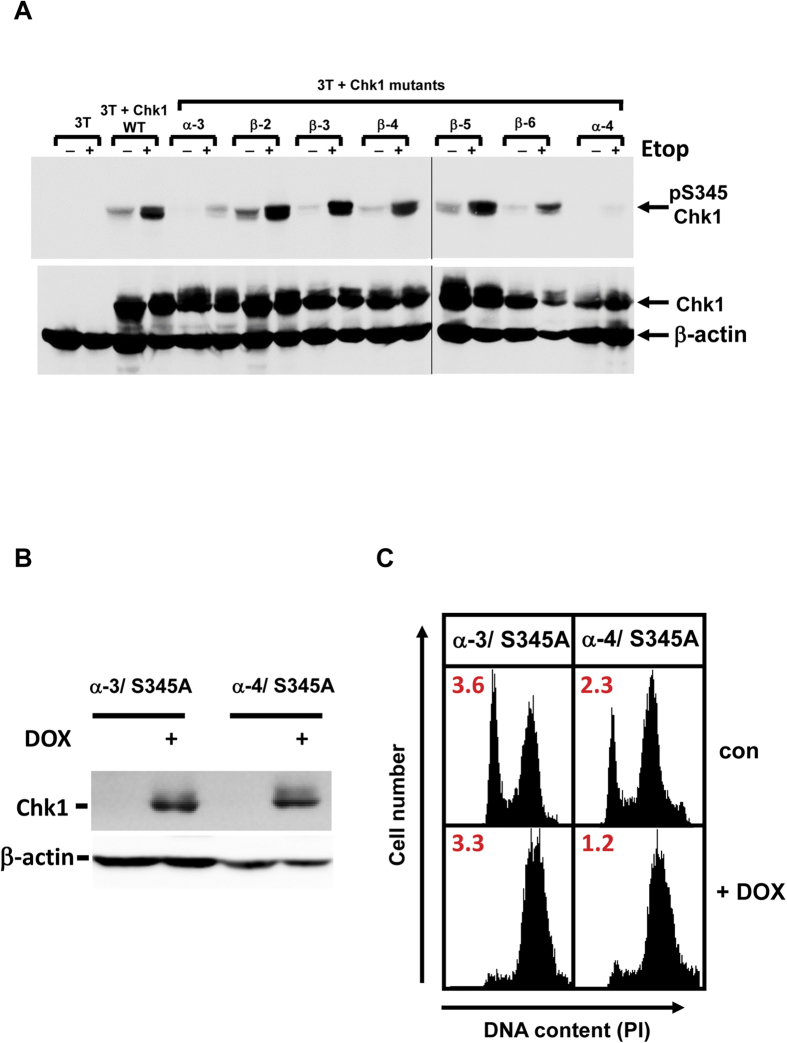
Chk1 KA1 mutants do not require S345 phosphorylation for biological activity. **A**) 3T cell cultures expressing Chk1 WT or the indicated mutants were treated for 16 hours with DOX to induce Chk1 expression then treated with etoposide or vehicle control for a further 6 hours. Samples were analysed by western blotting using a phospho-specific antibody that recognises Chk1 phosphorylated on serine 345 (pS345, upper panel) or total Chk1 (lower panel). β-actin serves as loading control. **B**) Secondary mutations of serine 345 to alanine (S345A) were introduced into Chk1 α-3 and α-4 to generate the double mutants Chk1 α-3/S345A and Chk1 α-4/S345A. 3T cell lines expressing each mutant were treated with DOX (50 ng/ml) or vehicle control for 16 hours, harvested, and analysed by western blotting (left panel) or flow cytometry for DNA content and mitotic index (right panel). β-actin serves as loading control. A complete analysis of individual cell cycle phases is presented in Supplementary Fig. 3B.

As shown in Fig. 5A, the basal level of S345 phosphorylation of WT Chk1 and in all of the Chk1-CA β-mutants was similar, whilst etoposide treatment induced a similar several-fold increase in each case. In comparison, the level of both basal and etoposide-induced S345 phosphorylation was significantly reduced for the Chk1 α-3 and α-4 mutants. These data indicate that whereas the Chk1-CA β-mutants can be phosphorylated normally by ATR in response to DNA damage this modification is diminished for Chk1 α-3 and Chk1 α-4. It is also clear from this analysis that none of the Chk1-CA mutants exhibit any spontaneous increase in the basal level of S345 phosphorylation that could account for their constitutive biological activity in the absence of induced DNA damage.

As a result, we next explicitly tested whether S345 phosphorylation was necessary for the biological activity of the Chk1-CA mutants as has previously been shown for WT Chk1^3^. To this end we introduced secondary mutations of S345 to alanine (S345A) in the context of the Chk1 α-3 and Chk1 α-4 mutants. The resulting double mutants Chk1 α-3/S345A and Chk1 α-4/S345A were then stably introduced into 3T cells to assess their effects on cell cycle progression. Strikingly, when induced with DOX the Chk1 α-3/S345A and Chk1 α-4/S345A double mutants induced cell cycle arrest in G2 phase as potently as their single mutant parents (Fig. 5C, Supplementary Fig. 3B), indicating that, in marked contrast to WT Chk1, modification of this critical regulatory site is dispensable for the biological activity of Chk1-CA mutants.

## Discussion

The biological functions of Chk1 have been explored in detail in recent years through a combination of genetic and molecular approaches. From such studies it has been established that Chk1 is the key effector of the G2 checkpoint and that this function is normally activated only when DNA damage is present^1^. Several studies have also established the critical importance of ATR-mediated S345 phosphorylation within the C-terminal regulatory region in triggering the checkpoint function of Chk1^2^,^3^,^4^, however the molecular mechanism through which this modification elicits activation has remained enigmatic. Attempts to understand this problem have been hampered by lack of insight into potential structural features within the C-terminal regulatory region combined with evidence that this region exerts both negative and positive effects on Chk1 function^11^.

Recently it was proposed that the C-terminal regulatory region of Chk1 contains a KA1 domain^12^, although no direct structural confirmation of this proposal was available. Here, we used the structural prediction algorithm FUGUE^18^ to analyse the Chk1 regulatory region. This analysis also predicted the presence of a KA1 domain in Chk1 with maximal similarity to that found in the plant stress kinase SOS2^13^. Interestingly, significant amino acid identity and similarity between the C-terminal regulatory domains of SOS2 and Chk1 was previously detected by conventional BLAST searching (Fig. 1A;^19^), although the significance of this finding has remained unresolved. The SOS2 KA1 domain plays a key role in regulation of the kinase by mediating both auto-inhibition and interactions with the positive and negative trans-regulators SOS3 and PP2C-family phosphatases respectively^13^.

Previous attempts to explore the Chk1 regulatory domain by mutagenesis in the absence of structural insights have, with few exceptions^11^,^25^,^26^, generated only loss-of-function mutants, indicating that such empirical mutations generally compromise some essential function residing within this region^11^,^12^. Here, we used the FUGUE-generated alignment of Chk1 with the structurally-defined SOS2 KA1 domain^13^ to target specific elements within the predicted Chk1 core KA1 domain by site-directed mutagenesis. The mutations we created were intended either to destabilise the overall KA1 fold by disrupting key helical elements (Chk1 α-3, Chk1 α-4), or to identify essential charged and hydrophobic amino acids located in the predicted β-sheets (Chk1 β2-β6).

Remarkably, none of these targeted Chk1 mutations resulted in loss-of-function; instead six of seven conferred a gain-of-function phenotype where inducible expression of the mutant proteins resulted in a profound G2 arrest in unperturbed cells. Given that Chk1 normally induces G2 arrest only under conditions of acute DNA damage, it is evident that these mutations confer constitutive biological activity on Chk1 (Chk1-CA). The sole exception to this pattern was mutant Chk1 β-2 which appears to be functionally indistinguishable from WT Chk1. That these targeted mutations confer a gain of biological function at such high frequency provides strong experimental support for the existence of a KA1 domain in Chk1. It also suggests that the putative KA1 structure plays a key role in restraining the biological activity of Chk1 in the absence of DNA damage.

We also explored the relationship between constitutive Chk1 biological activity, kinase catalytic activity, and the requirement for phosphorylation at S345, a modification catalysed by ATR and known to be essential for the activation of WT Chk1^3^,^4^,^10^. When Chk1-CA mutations α-3 and α-4 were combined with a “kinase-dead” mutation (K38R) the resulting double-mutants were biologically inert, clearly demonstrating that their biological gain-of-function phenotype depends on kinase catalytic activity. Consistent with this, Chk1-CA mutants also exhibited increased basal levels of kinase activity measured using an exogenous substrate (CDC25C). Although the increases in substrate-directed kinase activity were modest (2-6 fold), we note that previous studies have generally reported only small increases in kinase activity when WT Chk1 is activated in response to DNA damage^3^,^27^,^28^. It is known that Chk1 acts to block activation of the mitotic CDK1/B-cyclin complex to elicit G2 arrest under conditions of DNA damage, and that CDK1/B-cyclin activation is governed by a complex web of positive and negative feedback controls^29^. By acting on multiple interacting components such as CDC25 and Wee1 we presume that relatively modest increases in basal Chk1 activity can be sufficient to “tip the balance” and block CDK1 activation^29^.

We also observed a strong correlation between constitutive Chk1 biological activity and increased auto-phosphorylation as judged by altered electrophoretic mobility (Fig. 4B). We recently mapped threonines 378 and 382 (T378/T382) as sites of Chk1 autophosphorylation *in vitro* using biochemical techniques (N Morrice, unpublished results), and strikingly, the Chk1-CA mutants rapidly auto-phosphorylate these residues. T378 and T382 lie within the region of Chk1 predicted to correspond to the PP2C-binding (PPI) motif in SOS2. Interestingly, both residues lie within consensus Chk1 phosphorylation motifs (LxKxxT^378^ and MxRxxT^382^;^30^), and mutations between these residues in a putative PCNA-binding motif were previously shown to disable Chk1 biological function but to markedly enhance kinase catalytic activity^31^. These observations suggest that phosphorylation of T378/T382 within this putative “PPI” motif in Chk1 could have regulatory significance, although further work will be required to evaluate this and to identify all of the sites of auto-phosphorylation in Chk1-CA mutants.

Strikingly, we found that Chk1-CA mutants do not require phosphorylation of the essential S345 ATR site for biological activity. This was evident from two key findings; firstly, there was no increase in the basal level of S345 phosphorylation in the Chk1-CA mutant proteins that could account for their constitutive biological activity in the absence of DNA damage, and secondly, substitution of S345 with a non-phosphorylatable alanine residue did not impair G2 arrest induced by Chk1 α-3 and Chk1 α-4. It has previously been demonstrated that substitution of S345 with alanine renders WT Chk1 biologically non-functional^2^,^3^,^4^, indicating that KA1-targeted mutations effectively circumvent the need for this positive-regulatory modification. Taken together, these findings suggests that although S345 phosphorylation is required to activate WT Chk1 in response to DNA damage this modification is not obligatory for subsequent biological function as has often been assumed.

It is known that the C-terminal regulatory region of Chk1 can bind to and exert an inhibitory effect on the kinase domain^8^,^9^ and it has further been proposed that activation may occur via a de-repression mechanism that alleviates this inhibition^3^. It seems likely therefore that mutations that confer the Chk1-CA phenotype compromise the inhibitory function of the Chk1 regulatory domain without disturbing the less well-characterised positive function(s) that are also known to reside within this region^11^,^12^. Based on analogy with the KA1 domain of SOS2 we speculate that Chk1-CA mutations disrupt a critical regulatory protein-protein interaction, either between the regulatory domain and the kinase domain, or alternatively, with a trans-acting repressor molecule as proposed previously^3^.

**Figure 6:**
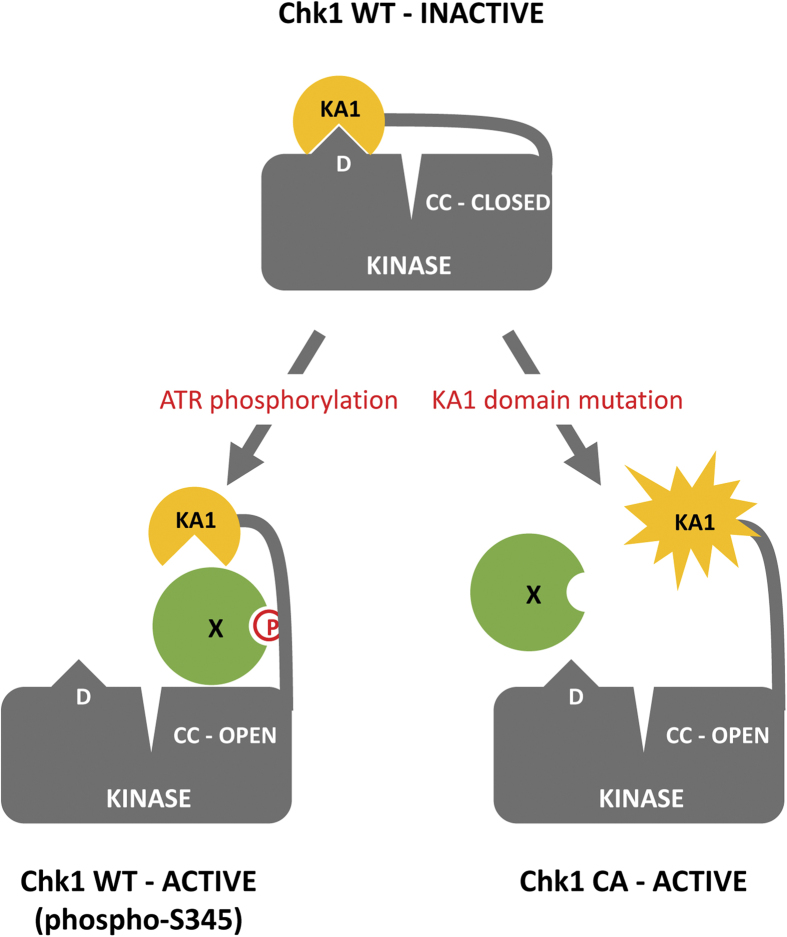
Model for the role of the putative KA1 domain in Chk1 regulation. In undamaged cells Chk1 exists primarily in an inactive, non-phosphorylated state where an intramolecular interaction between the putative KA1 domain and a hypothetical docking domain (D) has the effect of occluding the catalytic cleft (CC-CLOSED). In response to DNA damage, ATR is activated and phosphorylates the serine 345 (S345) regulatory site, thus creating a binding site for a trans-regulator (X). Binding of the trans-regulator to phospho-S345 activates Chk1 by dissociating the KA1-Kinase domain interaction and thus rendering the catalytic cleft accessible (CC-OPEN). By contrast, activating KA1 domain mutations dissociate the KA1-Kinase domain interaction and render Chk1 constitutively active (CC-OPEN) without the requirement for ATR-mediated S345 phosphorylation. Please see text for additional details.

Figure 6 depicts a hypothetical scenario, based partly on our observations described here, and partly on existing knowledge of the role of the KA1 domain in the regulation of SOS2^13^. We suggest that the KA1 domain docks against the Chk1 kinase domain and by so doing inhibits catalytic activity. We further propose that phosphorylation of the essential regulatory residue S345 by ATR in response to DNA damage creates a binding site for a transactivator molecule (X in Fig. 6), analogous to SOS3 in the case of SOS2^13^, whose physical interaction has the effect of dissociating the KA1-kinase domain and activating kinase catalytic activity. One potential candidate for “X” in the case of Chk1 would be 14-3-3 proteins, which are known to bind specifically to S345-phosphorylated Chk1^32^. We further suggest that Chk1-CA mutations dissociate the inhibitory intramolecular interaction by disrupting the structural integrity (*a*-mutants), or docking properties (β-mutants) of the KA1 domain, thus allowing kinase catalytic activity in the absence of S345 phosphorylation (Fig. 6). Further work, including structural studies, will be required to evaluate this model.

Finally, constitutively active kinases have been of great utility in dissecting the biological functions of complex signal transduction processes such as the MAPK and PI3K pathways. The availability of constitutively active forms of Chk1 opens up the prospect of analysing, and potentially manipulating, its effects on complex downstream checkpoint processes such as replication fork stabilization and homologous recombination in the absence of the pleiotropic and sometimes confounding effects of DNA damage or DNA synthesis inhibition.

## Materials and Methods

### Cell culture and treatments

3T DT40 cells and derived cell lines were grown in Dulbecco’s Modified Eagle’s Medium (DMEM; Invitrogen, Carlsbad, CA, USA) containing 10% tetracycline-free fetal bovine serum, 1% chicken serum, and 10 μM B-mercaptoethanol. 3T cells are a derivative of the Chk1 knockout DT40 cell line described previously^33^ containing a single, stably integrated copy of the pFRT/lacZeo acceptor plasmid of the Invitrogen “Flp-in” system together with the pcDNA6/TR plasmid encoding the Tet repressor. Chk1 mutants were cloned into the pcDNA5/FRT donor plasmid and co-transfected with the Flp recombinase-encoding plasmid pOG44 leading to stable integration of the mutants into the pFRT/lacZeo expression cassette under the control of a Tet-regulated promoter. Chk1 protein expression was induced by treating cultures with 50 ng/ml doxycycline (DOX) for between 16 hours and 4 days. To induce DNA damage cells were treated with 20 ng/ml etoposide for 6 hours.

### Site-directed mutagenesis

Chk1 mutations were generated using a “Quickchange” site-directed mutagenesis kit (Stratagene, La Jolla, CA, USA). Mutations were generated in the avian homologue of Chk1 but all of the amino acids that were altered as shown in Fig. 1A are identical in the sequence of human Chk1. The sequence of the mutagenic primers used to make the amino acid substitutions shown in Fig. 1B are available on request. All mutations were verified by sequencing prior to stable transfection into 3T cells and functional analysis.

### Flow cytometry

Cells were fixed in 70% ethanol in phosphate-buffered saline (PBS) or H_2_O overnight at 4 °C. Fixed cells were incubated phosphate-buffered saline containing 0.1% Tween 20 (PBT) with polyclonal anti-phospho serine 10 histone H3 antibodies (Santa Cruz Biotechnology, Santa Cruz, CA, USA; sc-8656) followed by fluorescein isothiocyanate-conjugated secondary antibody (Jackson Labs, Bar Harbor, ME, USA) for 1 hour. Cells were then counterstained in PBS containing 50 μg/ml propidium iodide and 250 μg/ml RNaseA for a further hour. Samples were analysed using a Becton Dickinson FACScan flow cytometer (BD Biosiences, Franklin lakes, NJ, USA) or MACSQuant Analyzer (Miltenyi Biotech, Lund, Sweden).

### Western blotting and kinase assays

Cells were lysed in ice-cold whole-cell extract buffer (WCE buffer^3^), or lysis buffer (LB^34^) resolved by SDS-PAGE gel electrophoresis, and analysed by western blotting using antibodies recognising total and S345-phosphorylated Chk1 as described previously^3^,^34^. For Chk1 kinase assays WT and mutant Chk1 proteins were immunoprecipitated from extracts prepared using WCE buffer using an antibody recognising the C-terminus of Chk1^3^. Immunoprecipitates were washed three times with WCE buffer and twice with Chk1 kinase buffer as described previously^3^. For conventional Chk1 kinase assays 200 ng of soluble GST-CDC25 (amino acids 250-256) substrate in 30 μl of Chk1 kinase buffer was added and the reaction initiated by addition of ATP. Reactions were incubated at 30 °C for between 10 and 30 minutes after which the reactions were terminated by addition of SDS-PAGE sample buffer, resolved by SDS-PAGE gel electrophoresis, and analysed by western blotting using a phospho-specific antibody recognising CDC25 phosphorylated at serine 216 (p216; Cell Signalling Technology, Cat no. 9528). Membranes were subsequently re-probed using an antibody recognising total Chk1 (Santa Cruz Biotechnology, Cat no. sc-8408). CDC25 pS216 signals were quantified by densitometry using ImageQuant software and normalised to the amount of Chk1 protein immunoprecipitated in each reaction. For auto-phosphorylation assays WT and mutant Chk1 proteins were immunoprecipitated, washed, and incubated in Chk1 kinase buffer plus or minus ATP in the absence of GST-CDC25 substrate. Reactions were terminated by addition of SDS-PAGE sample buffer, resolved by SDS-PAGE gel electrophoresis, and analysed by western blotting using an antibody recognising either total Chk1 (Santa Cruz Biotechnology, Cat no. sc-8408), or a phospho-specific antibody generated using a di-phosphorylated peptide spanning threonine 378 and threonine 382 (T378/T382) of avian Chk1. The mapping of these sites of auto-phosphorylation and characterisation of this phospho-specific antiserum will be described elsewhere.

## Additional Information

**How to cite this article**: Gong, E.-Y. *et al.* KA1-targeted regulatory domain mutations activate Chk1 in the absence of DNA damage. *Sci. Rep.* **5**, 10856; doi: 10.1038/srep10856 (2015).

## Supplementary Material

Supplementary Information
