# Supplementary material for: KA1-targeted regulatory domain mutations activate Chk1 in the absence of DNA damage
Source: Sci Rep. 2015 Jun 3;5:10856. doi: 10.1038/srep10856 (PMC4454167; doi:10.1038/srep10856)

Supplementary Data

KA1-targeted regulatory domain mutations activate Chk1 in the absence of DNA damage

Eun-Yeung Gong[3], Veronique AJ Smits[2] Felipe Fumagallo[2], Desiree Piscitello[3], Nick Morrice[3], Raimundo Freire[2], and David A Gillespie[1, *]

[1]Instituto de Tecnologías Biomédicas, Centro de Investigaciones Biomédicas de Canarias, Facultad de Medicina, Campus Ciencias de la Salud, Universidad de La Laguna, La Laguna 38071, Tenerife, Spain

[2]Unidad de Investigación, Hospital Universitario de Canarias, Instituto de Tecnologías Biomédicas, Ofra s/n, La Cuesta, La Laguna 38320, Tenerife, Spain

[3]Beatson Institute for Cancer Research, Garscube Estate, Switchback Road, Glasgow G61 1BD, U.K.

*Corresponding author.

Email: dgillesp@ull.es
Tel: +34 922 34111
Fax: +34 922 647112

Running title: Chk1-activating mutations

Legends to Supplementary Figures 1-3

Supplementary Fig. 1

A) Expression of Chk1 KA1 mutants +/ - DOX and comparison with endogenous Chk1 in DT40 cells

The indicated cell cultures were treated with DOX (50ng/ ml) or solvent control for 16 hours prior to harvest and analysis of Chk1 protein expression. Cell extract from wild-type DT40 cells is included for comparison. The levels of exogenous WT and mutant Chk1 protein expression were approximately 3-5 times greater than the level of endogenous Chk1. β-actin serves as loading control.

B) G2 arrest induced by Chk1-CA mutants is associated with increased CDK1 tyrosine 15 (Y15) phosphorylation

The indicated cell cultures were treated with DOX (50ng/ ml) or solvent control for 16 hours prior to harvest and analysis of phospho-Y15 CDK1 by western blotting. β-actin serves as loading control.

Supplementary Fig. 2

Chk1 KA1 mutants induce cell cycle arrest in G2

3T cell cultures expressing Chk1 WT or the indicated mutants were treated for 16 hours with DOX or vehicle control, harvested, and analysed by flow cytometry for DNA content and phospho-serine 10 histone H3 (pH3) as shown in Fig. 3. The percentage of G1, S, G2, and M phase cells was quantified by gating the two-dimensional plots.

Supplementary Fig. 3

A) Chk1 catalytic activity is essential for G2 arrest induced by Chk1-CA mutants

3T cell cultures expressing the indicated mutants were treated for 16 hours with DOX or vehicle control, harvested, and analysed by flow cytometry for DNA content and phospho-serine 10 histone H3 (pH3) as shown in Fig. 4C. The percentage of G1, S, G2, and M phase cells was quantified by gating the two-dimensional plots.

B) Chk1 KA1 mutants do not require S345 phosphorylation for biological activity

3T cell cultures expressing the indicated mutants were treated for 16 hours with DOX or vehicle control, harvested, and analysed by flow cytometry for DNA content and phospho-serine 10 histone H3 (pH3) as shown in Fig. 5C. The percentage of G1, S, G2, and M phase cells was quantified by gating the two-dimensional plots.

Supplementary Fig. 1

A

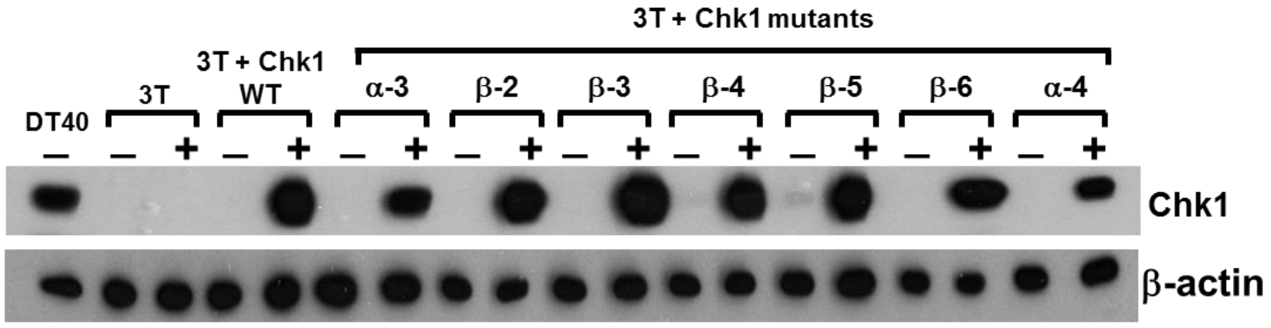

B

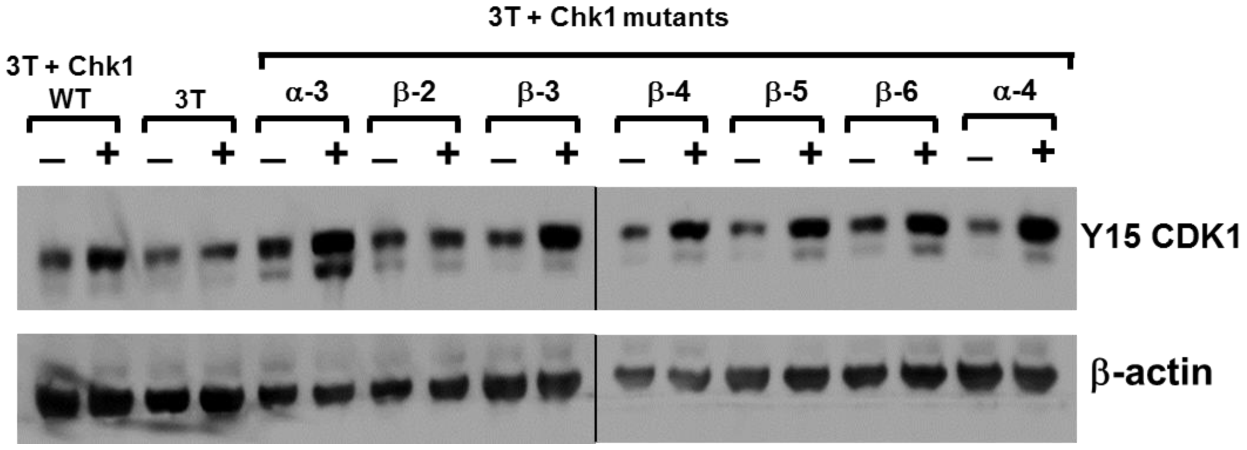

Supplementary Fig. 2

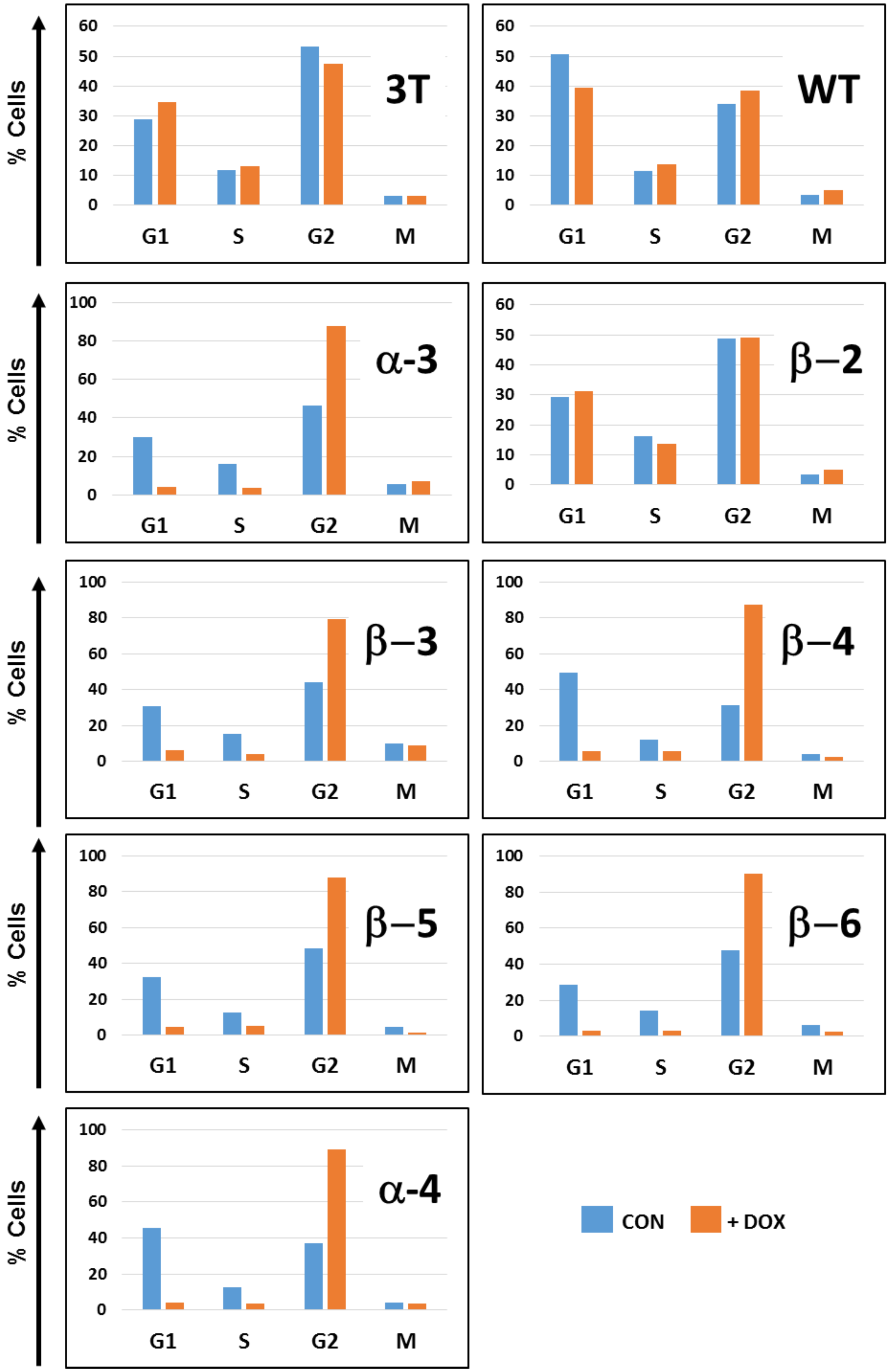

Supplementary Fig. 3

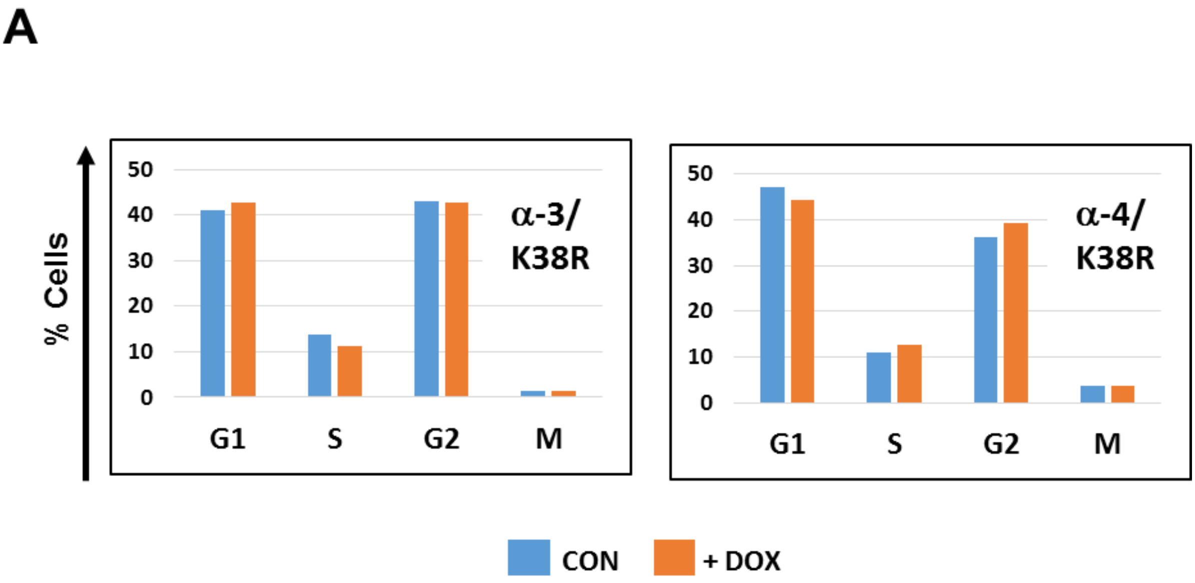

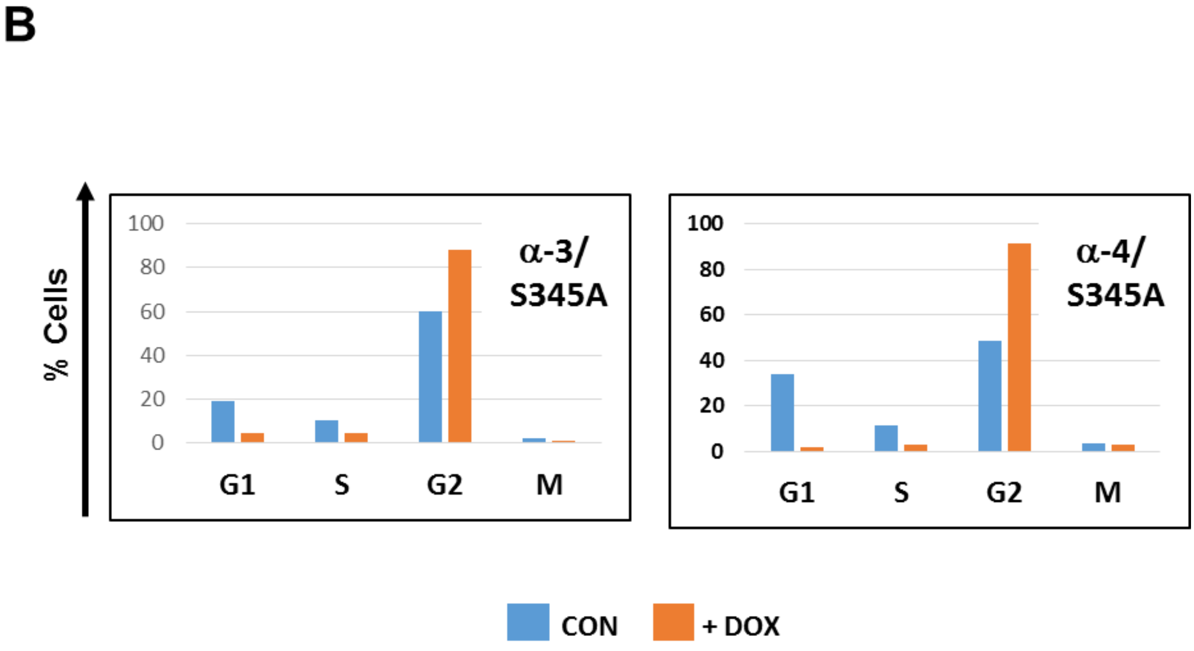

Supplement: Supplementary Information [file srep10856-s1.pdf]
